# Systematic review and meta-analysis of serum total testosterone and luteinizing hormone variations across hospitalized Covid-19 patients

**DOI:** 10.1038/s41598-024-53392-7

**Published:** 2024-02-02

**Authors:** Stefano Salciccia, Martina Moriconi, Vincenzo Asero, Vittorio Canale, Michael L. Eisenberg, Frank Glover, Federico Belladelli, Nicolas Seranio, Satvir Basran, Ettore De Berardinis, Giovanni Di Pierro, Gian Piero Ricciuti, Benjamin I. Chung, Alessandro Sciarra, Francesco Del Giudice

**Affiliations:** 1 Department of Maternal Infant and Urologic Sciences, “Sapienza” University of Rome, Policlinico Umberto I Hospital, 00161 Rome, Italy; 2 Department of Urology, Stanford University School of Medicine, Stanford, CA 94305 USA; 3 Emory University School of Medicine, Emory University, Atlanta, GA 30322 USA; 4 Division of Experimental Oncology/Unit of Urology, URI, IRCCS San Raffaele Hospital, Milan, Italy

**Keywords:** Immunology, Microbiology, Endocrinology, Risk factors, Urology

## Abstract

A growing body of evidence suggests the role of male hypogonadism as a possible harbinger for poor clinical outcomes across hospitalized Covid-19 patients. Accordingly, we sought to investigate the impact of dysregulated hypothalamic-pituitary–gonadal axis on the severity of the clinical manifestations for hospitalized Covid-19 patients matched with healthy controls through a systematic review and meta-analysis. Databases were searched from inception to March 2022. A standardized mean difference (SMD) meta-analysis focused on hospitalized Covid-19 patients and healthy controls was developed for studies who reported total testosterone (TT) and luteinizing hormone (LH) levels at hospital admission. Overall, n = 18 series with n = 1575 patients between 2020 and 2022 were reviewed. A significant decrease in SMD of TT levels in Covid-19 patients compared to paired controls was observed (− 3.25 nmol/L, 95%CI − 0.57 and − 5.93). This reduction was even more consistent when matching severe Covid-19 patients with controls (− 5.04 nmol/L, 95%CI − 1.26 and − 8.82) but similar for Covid-19 survivors and non-survivors (− 3.04 nmol/L, 95%CI − 2.04 and − 4.05). No significant variation was observed for serum LH levels across studies. Patient related comorbidities, year of the pandemic, and total lymphocyte count were associated with the observed estimates. TT levels may be a useful serum marker of poor outcomes among Covid-19 patients. These findings may support the development of ad-hoc clinical trials in the Covid-19 risk-group classification and subsequent disease monitoring. The interplay between TT and immune response should be evaluated in future researches.

## Introduction

The severe acute respiratory syndrome coronavirus 2 (SARS-CoV-2), causing Covid-19 infection, represents a severe worldwide health emergency^1^. To date, a large volume of clinical data has been published, and numerous risk factors for adverse clinical outcomes and mortality in patients with Covid-19 disease have been identified^2^. Among the risk factors identified, male sex and older age represent important risk factors for severe course of Covid-19. These trends support the hypothesis that both sex-differences in immune response and hormonal constitution may play a crucial role on the susceptibility to infection and in the severity of the clinical course^3^. Sex-specific differences in immune responses are well-known phenomena described in the scientific literature, and also within the context of the Covid-19 pandemic^4^. The two most important factors accounting for the sex-bias in immunity are genetics and sex hormones, with the most supported theory suggesting that oestrogens have a protective role for women, whereas androgens promote worse clinical results in men^5^. Moreover, interest in testosterone in the context of SARS-Cov-2 infection was renewed by the observation that SARS*-*CoV*-*2 viral entry into host cells is dependent on angiotensin converting enzyme 2 (ACE2) and transmembrane serine protease 2 (TMPRSS2), which have been found to be expressed in human lungs and other tissues, including prostate and testis^6^. TMPRSS2 plays a crucial role in the entry of SARS-CoV-2 virus into the respiratory epithelial cells leading to COVID-19 disease^7^. TMPRSS2 is expressed in prostate epithelium and is regulated by the androgen receptor (AR). In addition to these known mechanisms, the role played by androgens in the pathophysiology of Covid-19 infection appears to be multifaceted, involving both viral replication and immunological response to the virus. In this context, it was hypothesized that low plasmatic testosterone levels can be determinant as for infection outcome and SARS-CoV-2 replication as well, through the modulation of intracellular Calcium ([Ca2^+^]i) homeostasis in host cells^8^. Moreover testosterone levels seem to play a crucial role in activating the immune response to infection, androgens immunosuppressive effect can play a decisive role in the more advanced phase of the disease, characterized by a dysregulated immunological response to systemic inflammation. Despite these findings that led researchers to propose androgen deprivation therapy (ADT) or testosterone replacement therapy in patients with Covid-19, the role of testosterone serum levels in the clinical course of the disease remains controversial, mainly due to the complex actions exerted by androgens, including the modulation of the immune response^9^. Furthermore, late onset hypogonadism is a frequent syndrome in older age, and it includes clinical manifestations like obesity, insulin-resistance and cardiovascular diseases, the so-called “inflammaging”, which typically predispose to COVID-19 infection and worse clinical outcomes^10–12^. Several studies have identified low testosterone levels as a significant risk factor for adverse clinical outcomes in the current Covid-19 pandemic. Moreover a recent meta-analysis by Corona et al. evaluated the andrological consequences of COVID-19 both on seminal and hormonal parameters, reporting that low testosterone levels, detected in the acute phase of the disease, is associated with an increased risk of admission to the Intensive Care Unit or death^13^. However, despite these findings, the specific role of TT in the pathophysiology of Covid-19 infection needs to be further investigated. Based on these considerations, the primary aim of this systematic review and meta-analysis is to describe serum total testosterone levels (TT) and luteinizing hormone levels (LH) in patients with Covid-19 infection and their associations with infection severity. Secondary, the aim is to investigate the role of patient available comorbidities or inflammatory/haemato-chemical variables retrieved to TT by meta-regression analysis.

## Results

### Search results

**Figure 1:**
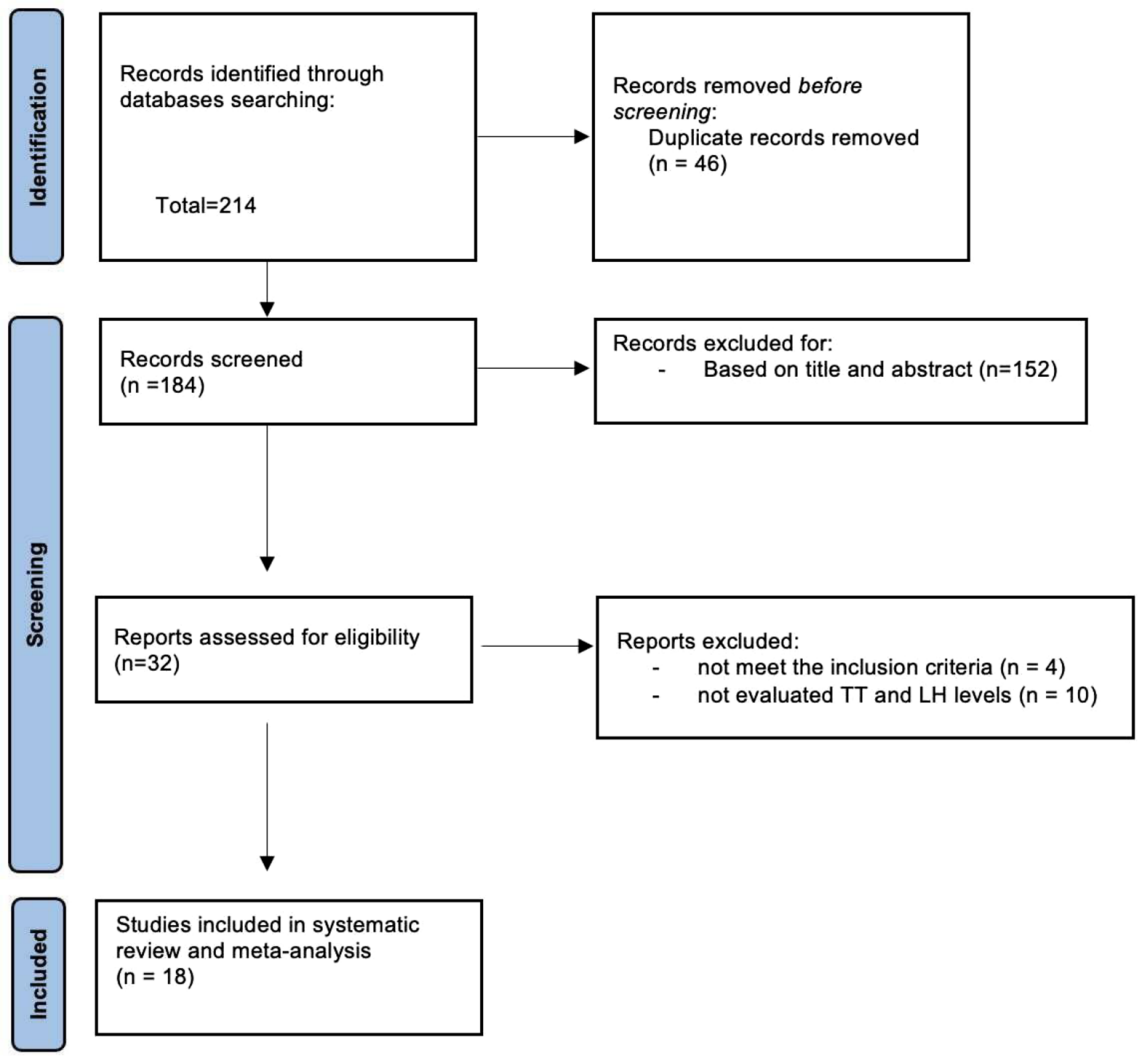
PRISMA flow diagram. TT: Testosterone; LH: Luteinizing Hormone.

The search strategy identified 214 studies and 6 additional papers were identified through other sources for a total of 220 studies. After removing the duplicates, 184 studies were screened of which 152 were excluded based on title and abstract. Full text of the 32 selected studies was obtained. 14 studies were excluded: 4 studies were conducted on animal models, 10 didn’t evaluate TT and LH levels. The PRISMA flow diagram is presented in Fig. 1. Complessively, 18 records fulfilled the inclusion criteria and were included in the final analysis^14–31^.

### Location, design, and characteristics of the studies population

Eighteen studies met the inclusion criteria and were included in this analysis, overall 1,575 male patients with Covid-19 infection and 886 male controls. According to the severity of the disease, 823 patients were classified as presenting moderate disease and 500 as having severe disease. Patients enrollment ranged between 2020 and 2022. 15 studies^14,17–22,24–31^ exhibited a prospective cohort design and 3 studies reported a retrospective cohort design^15,16,23^. The studies included were conducted in Turkey^14,17,21,22,25,29^ Italy^15,16,26–28^ China^24,30,31^ USA^18^, Greece^20^, Russia^19^, Austria^23^. Among the studies included, 11 were controlled^16,17,19–21,24,25,28–30^. Collected data included testosterone levels ^14–31^, LH levels^14,17,19,22–24,28–30^, IL-6 levels^15,23,26–28,30,31^, lymphocytes count^14–16,23,24,26–28,30,31^, D-dimer levels^14–16,20,24,26,27,31^. The characteristics of the 18 studies included are presented in Table 1.

**Table 1:** Clinical and Demographic characteristics of the studies assessing serum total testosterone (TT) and luteinizing hormone levels (LH) enrolled in the systematic review and meta-analysis.

Study Author	Year	Study Design	Country	PTS control (n)	PTS covid (n)	PTS moderate (n)	PTS severe (n)	TT control	TT covid	LH control	LH covid
Apaydin	2021	Prospective	Turkey		81	54	27		7.63		7.01
Beltrame	2022	Retrospective	Italy		68	56	12				
Camici	2021	Retrospective	Italy	24	24	24		10.41	5.23		
Cinislioglu	2021	Prospective	Turkey	92	358	153	205	11.94	6.21	3.75	7.3
Dhindsa	2021	Prospective	USA		90	24	66		3.48		
Enikeev	2022	Prospective	Russia	44	44	44		13.5	7.3	3.08	3.34
Ilias	2021	Prospective	Greece	29	65		55	8.53			
Karkin	2021	Prospective	Turkey	70	70	48		3.46	2.97		
Koc	2021	Prospective	Turkey	21	21	21		12.15	10.06	3	3.1
Lanser	2021	Retrospective	Austria		155				5.67		6.49
Ma	2020	Prospective	China	273	119	103	16	15.23	14.92	3.46	6.45
Okcelik	2020	Prospective	Turkey	20	24	24		11.25	12.29		
Rastrelli	2020	Prospective	Italy		31	21	6				
Salciccia	2021	Prospective	Italy		29	9	20		16.17		
Salonia	2021	Prospective	Italy	281	286	174	51	10.64	2.74	4.17	4.8
Temiz	2020	Prospective	Turkey	10	10	10		10.06	3.92	4.46	2.98
Xu	2020	Prospective	China	22	39	20	19	13	13.64	8.05	6
Zheng	2021	Prospective	China		61	38	23				

*Pts* patients, *TT* testosterone, *LH* luteinizing hormone.

### Serum TT levels variation across Covid-19 patients and healthy controls

**Figure 2:**
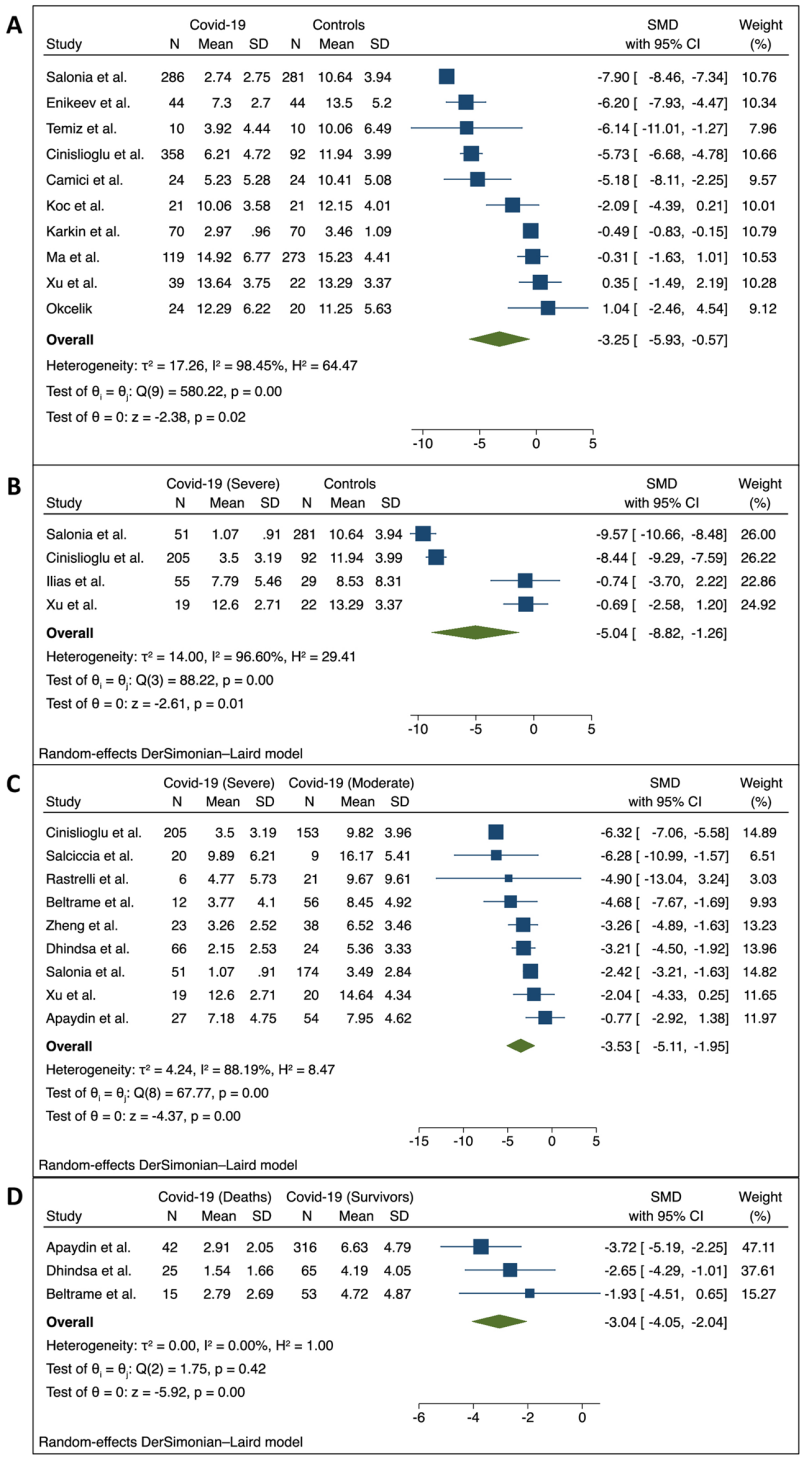
Forest plot for Standardized Mean Difference (SMD) across studies presenting serum Total Testosterone (TT) in Covid-19 male patients vs. male control (**A**), clinically severe Covid-19 patients vs. control (**B**), clinically severe Covid-19 vs. clinically moderate Covid-19 patients (**C**) and, Covid-19 related deaths vs. Covid-19 survivor patients (**D**).

Out of n = 18^14–31^ studies encompassed in the systematic review, n = 10^16,17,19,21,22,24,25,28–30^ were further meta-analytically compared with regards to mean TT levels variations between Covid-19 and control populations. Of note, at this preliminary assessment, there was considerable heterogeneity across each single study with I^2^ 98.45%, Q (9): 580.22, *p* < 0.00. Publication bias was initially assessed by Galbright and Funnel plot (Suppl. Fig. 1). Inspection of both plots suggested that there was no small-study effect with the smaller studies tending to have higher SMD variability, suggesting absence of publication bias (Egger test, *p* = 0.96). Additionally, the “Trim and Fill” method suggested that no studies would have needed to be included to remove residual asymmetry from the funnel plot. The main contribution to study heterogeneity was indeed identified by sub-group analysis summarized in Suppl. Fig. 2. Interestingly, year of publication which in this case is directly associated with the progression of the Covid-19 pandemic exhibited a significant ascending influence on SMD observed, while no further study design nor cumulative sample size of patients accrued was associated with the observed heterogeneity. The effect of publication year was further highlighted at cumulative meta-analysis sorted by year, where increasing variations in SMD of TT levels across the studies were observed (Suppl. Fig. 2). However, as per sensitivity analysis, the leave-one-out analysis suggested acceptable variation along the cumulative SMD observed when clustering the data by publication year. According to predefined random-effects model, we found a significant reduction in the SMD of testosterone levels in Covid-19 patients compared to controls (− 3.25, 95%CI − 5.93, − 0.57, *p* = 0.00, I^2^ = 98.45%). The difference was even higher when examining men with severe Covid-19 (− 5.04, 95%CI − 8.82, − 1.26, *p* = 0.00, I^2^ = 96.60%) (Fig. 2).

Additionally, within the Covid-19 population, we assessed patients stratified by severity of clinical manifestations. As further evidence, compared with patients exhibiting moderate Covid-19 symptoms, patients with more severe sequelae exhibited lower levels of testosterone showing a slight yet significant absolute higher SMD (− 3.53 nmol/L, 95%CI − 5.11, − 1.95, *p* = 0.00, I^2^ = 88.19%).

This was corroborated across the n = 3^14,15,18^ experiences which reported outcomes stratified according Covid-19 survivors and non-survivors, confirming a similar overall decrease in SMD serum TT levels (− 3.04, 95%CI − 4.05, − 2.04, *p* = 0.42, I^2^ = 0.00%) (Fig. 2).

Finally, in order to explore the remaining heterogeneity observed, we investigated the role of patient available comorbidities or inflammatory/haemato-chemical variables retrieved to the SMD estimates by meta-regression analysis. As expected, there was a direct correlation between increasing BMI of the Covid-19 population and the SMD observed across the studies (Coeff. 2.48, SE: 1.15; *p* = 0.033; Suppl. Fig. 3). Moreover, the sole haemato-chemical confounder significantly associated with TT variation was the absolute lymphocyte count depicting an inverse trajectory with the SMD observed across the studies (Coeff. − 7.37, SE: 2.19; *p* = 0.001; Suppl. Fig. 3).

### Serum LH levels variation across Covid-19 patients and healthy controls

Regarding LH levels, only n = 7^17,19,22,24,28–30^ studies reported the needed information to further compare the overall populations and the sub-groups assessing the outcomes according to the severity of Covid-19 clinical manifestations. A considerable heterogeneity was documented also in this setting with I^2^ 95.60%, Q(6): 136.32, *p* < 0.02. Additionally, the inspection of both Galbright and Funnel plots suggested that there was a significant small-study effect with the smaller studies tending to have higher SMD variability, suggesting the existence of higher risk of bias among the publications assessed (Egger test, *p* = 0.035). However, the “Trim and Fill” method suggested that only n = 1 study would have needed to be included to remove residual asymmetry from the Funnel plot (Suppl. Fig. 4). This preliminary finding was further confirmed at sub-group analysis where the size of the sample analysed was significantly associated with greater variability in the pooled SMD yet not being influenced by the publication year like previously observed (Suppl. Fig. 4).

**Figure 3:**
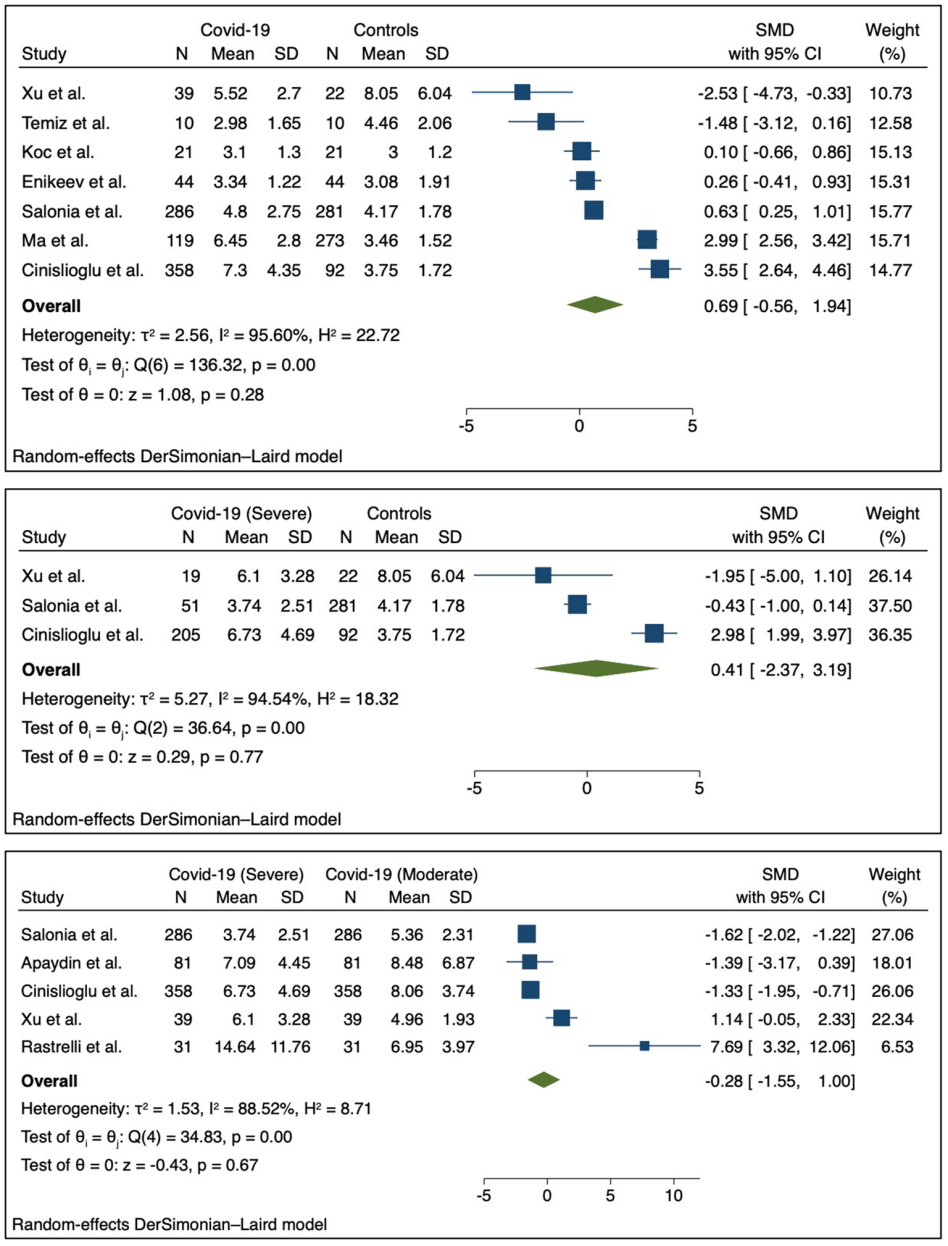
Forest plot for Standardized Mean Difference (SMD) across studies presenting serum Luteinizing Hormone (LH) levels in the Covid-19 patients vs. control (**A**), clinically severe Covid-19 patients vs. control (**B**), and clinically severe Covid-19 vs. clinically moderate Covid-19 patients (**C**).

In contrast to TT levels, the effect on cumulative LH variations was non-significant nor clinically relevant between Covid-19 cases and matched controls (SMD: 0.69, 95%CI 0.56, − 1.94, *p* = 0.00, I^2^ = 95.60%) ranging along the null-effect line from 3.55, 95%CI 2.64, − 4.46, to − 2.53, 95%CI − 4.73, − 0.33 in the study of Cinislioglu et al.^17^ and Xu et al.^30^ respectively. This was also true when comparing controls with the n = 3^17,28,30^ studies with more severe Covid-19 population (SMD: 0.41, 95%CI − 2.37, 3.19, *p* = 0.00, I^2^ = 95.54%) (Fig. 3).

Furthermore, at sensitivity analysis through leave-one-out assessment there was no single study effect size which would have significantly impaired the observed pooled results if omitted from the analysis (Suppl. Fig. 5).

At meta-regression on quantitative variables available, the relative percentage of Covid-19 patients with active smoking status was the sole factor influencing the pooled SMD estimate for serum LH levels (Coeff. − 0.08, SE: 0.01; *p* = 0.001; Suppl. Fig. 6).

Finally, when sub-analyzing within the Covid-19 severity of clinical manifestations, there was no significant or clinically relevant SMD across the n = 5 studies included^14,17,26,28,30^ − 0.28 (95%CI − 1.55, 1.00, *p* = 0.00, I^2^ = 88.52%).

## Discussion

A growing body of evidence has demonstrated older age, male sex, and medical comorbidities as being risk factors for Covid-19 mortality^1^. In particular, male sex and older age were found to be significant determinants for severe SARS-CoV-2 phenotype, supporting the hypothesis that hormonal constitution may be a key factor for both Covid- 19 susceptibility and acute respiratory distress syndrome (ARDS) development. Here we report serum testosterone variation according to the presence of the disease and severity of presentation. In this meta-analysis of 18 studies, reflecting 1575 patients with moderate and severe Covid-19, we evaluate serum testosterone levels according to stage of the disease. The results show significantly decreased levels of testosterone in moderate vs. severe patients (SMD: − 3.48, 95% CI − 4.85, − 2.11, *P* < 0.001, I^2^: 84.99%) as well as between overall cases of Covid-19 and controls (SMD: − 3.24, 95% CI − 5.31, − 1.17, *p* < 0.002, I^2^: 97.32%). Moreover, this data is also confirmed by matching patients that survived vs died of Covid-19 (SMD: − 2.996, 95% CI − 4.00–1.98, *p* < 0.0001, I^2^: 51.72%). Although the descriptive nature of our study does not allow us to state with certainty that hypogonadism represents a clinical risk factor, we have observed a progressive reduction in testosterone values according to the stage of the disease, suggesting that TT should be assessed in patients with Covid-19 infection.

Among the studies included in the meta-analysis, only 4^14,15,23,28^ analysed low TT levels as a risk factor for hospitalized patients mortality or need of intensive care unit, evidenced by an odds ratio (OR) ranging between 0.64 and 0.74, respectively according to Apaydin et al.^14^ and Beltrame et al.^23^. Importantly, the exact pathophysiological role of androgens in the context of Covid-19 disease still remains controversial^32–35^.

Based on these results, we can formulate some hypotheses to explain the potential importance of low TT levels in terms of sex differences in Covid-19 severity. Firstly, as observed for many other severe illnesses, low TT levels may simply represent a marker of illness severity due to dysregulation of the hypothalamic-pituitary–gonadal axes^36–38^. Secondly, it has also been hypothesized that viral tropism for Leydig and Sertoli cells through the TMPRSS and ACE2 receptors would lead to a functional gonadal dysregulation, and therefore hypogonadism, which ultimately could lead to a worse course of the disease through a dysregulation of the immune response mediated by androgens^5,39,40^. To further explore this last hypothesis, we analyzed LH levels from cases and controls^17,19,22,24,28,30^ according to severity of the disease (moderate vs severe disease). Interestingly, we do not observe significant differences in LH levels either between cases and controls or between different disease stages. The results of this analysis, which encompasses 877 cases of Covid-19 and 743 controls, delineate primary hypogonadism, as a consequence of viral involvement of the testis during the course of the disease, unlikely. These results are consistent with some studies that failed to demonstrate that SARS-CoV-2 infection has an impact on male gonadal function in contrast to other viruses known as Mumps virus or HIV^41,42^. In this context, a recent and exhaustive meta-analysis by Corona G. et al. gives inconclusive results in demonstrating the presence of the virus at the seminal level, suggesting however that it could be identified in the acute phases of the disease^43^. Based on our results, we may formulate the hypothesis that the low TT observed can probably be the consequence of the generalized inflammation that characterizes Covid-19 disease due to the dysregulation of the hypothalamic-pituitary-peripheral axes^38^. However, for the same reason, linked to the dysregulation of the hypothalamic-pituitary–gonadal axis, the finding of normal LH values in correlation with low testosterone values does not allow us to exclude a direct involvement of the testicle by the virus during the course of the disease. Given the important clinical implications both on fertility and sexual transmission, this possibility should be evaluated in dedicated studies.

Although the testosterone values may simply represent a marker of disease severity, we cannot exclude that the state of hypogonadism represents an important risk factor for adverse clinical outcomes, through the loss of the immunomodulating role of androgens^5,40^. In this context, differences between male and female immune responses are well known, which demonstrates that genetics and sex hormones are important for the immune response^3,4^. Moreover in the context of Covid-19 pandemic, gender differences in immune response gained even more relevance since the two entry routes of the virus, such as TMPRSS2 and ACE2, appear regulated by androgens. The androgen-driven overexpression of these viral co-receptors may augment cellular entry and replication of SARS-CoV-2, thereby mediating increased infection rate and COVID-19 severity in men. This observation has led several researchers to hypothesize a protective role for androgen deprivation therapy (ADT), however with conflicting results; on one hand some studies demonstrate a protective effect on the susceptibility and severity of the infection, on the other hand, others fail to confirm the same results^44,45^. These conflicting outcomes suggest that the role of androgens may be more complex and go beyond the receptor-linked mechanism. Past studies demonstrate that gender has a significant impact on the outcome of infections and it is associated with underlying differences in immune response to infection and vaccines^46,47^. The exact mechanism by which androgens modulate the immune response is an evolving field of research. Evidence from unrelated studies points to an immunosuppressive role of testosterone on different components of the immune system, and also suggests a role of testosterone in the different phases of the immune response as well as a link between testosterone and the different cells involved in the immune response^48^.

**Figure 4:**
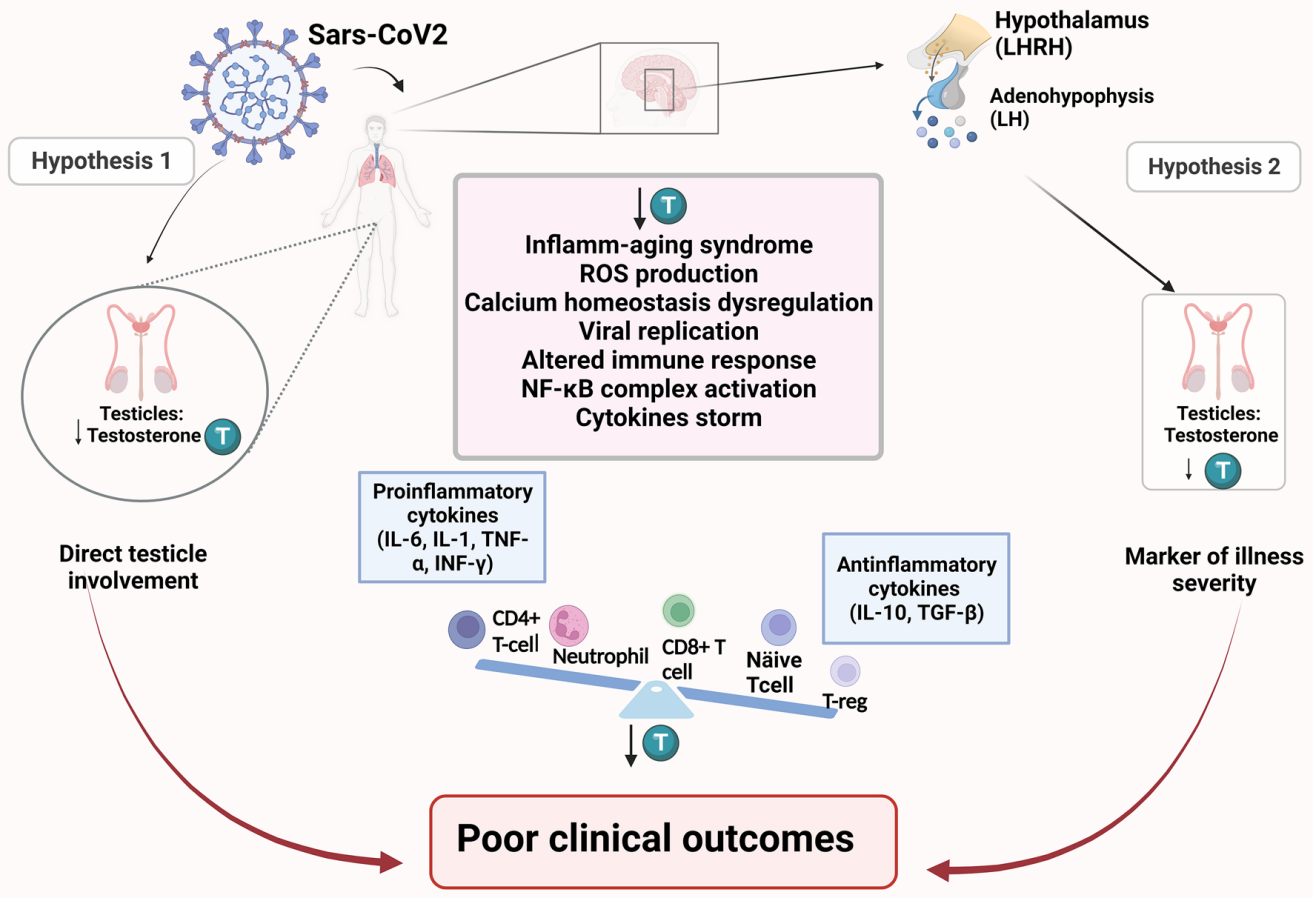
Pathogenetic hypothesis of COVID-19 infection and interplay between hypogonadism and immune response. Hypothesis 1. Sars-CoV-2 viral infection. The virus may present direct testicles cell tropism (such as for Leydig cells), some authors hypthesized a direct detrimental action on testosterone secretion, hence proposing a primary hypogonadism-like condition; Hypothesis 2. Hypogonadism may be a consequence of sexual axis involvement (hypothalamic–pituitary–adrenal axis) after Sars-CoV-2 infection. In patients presenting worse outcomes, hypogonadism may be the key to unfavourable events, encompassing altered immune response, leading to poor outcomes. Abbreviations: T testosterone; ROS Reactive Oxygen Species; NF-*k*B nuclear factor kappa-light-chain-enhancer of activated B cells. Created with BioRender.com.

Therefore, the role of androgens in modulating the immune response to infections is known and has become relevant again in the context of the covid-19 pandemic: Takahashi T. et al. in a series of 98 patients with Covid-19 infection note that male patients have higher plasma levels of innate immune cytokines such as IL-8 and IL-18 along with more robust induction of non-classical monocytes and a poor T cell response negatively correlate with patients' age^49^. However, there is limited scientific evidence concerning the role of androgens in determining this immune phenotype. Interestingly, it has been hypothesized that testosterone and DHT bind to androgen receptors on T lymphocytes, impairing T lymphocyte activation, and inhibit Th1 differentiation and interferon-gamma (IFN-γ) production, therefore resulting in a greater susceptibility to infection and severity of disease in men compared to women^50^. In addition to this mechanism, it has recently been highlighted that androgen suppression of TYK2 (member of the Janus Kinase, *JAK*, family of genes) signalling in T lymphocytes may be an important determinant of COVID-19 outcome^51^. However, it has also been supposed that the immunosuppressive effect of androgens may be crucial in the advanced phases of the disease characterized by a dysregulated immune response. This fact corroborates our results, observing that low TT levels are associated with worse clinical outcomes. Given the conflicting findings, we can speculate that the role of testosterone in the course of the disease may be twofold, suggesting the need to be further investigated. We conducted a meta-regression analysis with the aim to go through the complex relationships between testosterone levels, inflammatory markers and some actors of the immune response. Among the studies included in the meta-analysis, 7^14,16,18,23,26,27,31^ observe an inverse and statistically significant correlation between testosterone and IL-6 values and other inflammatory markers such as D-dimer, CRP, IL-1, LDH. While four studies^14,15,26,31^ observe a positive correlation between TT levels and lymphocyte count, suggesting that testosterone may have an important role in modulating the immune response to infection. To explore this evidence, in our meta-analysis we included data on several available inflammatory markers, and in the meta-regression analysis we can observe that the only haemato-chemical confounder significantly associated with TT variation was the absolute lymphocyte count, depicting an inverse trajectory with the SMD observed across the studies (Suppl. Fig. 3). The result of the meta-regressions supports the hypothesis that androgens can modulate the lymphocyte response to infection. On the other hand, it is recognized that lymphocytes express the androgen receptor and that low testosterone levels can lead to an altered immune response^52^. Our results are consistent with those observed by Zheng et al.^31^. They found that serum testosterone level was positively correlated with lymphocyte count with a correlation coefficient of 0.522 (*P* < 0.05). Furthermore, there are several studies supporting the role of TT in modulating the lymphocytes response: Page et al. observed that testosterone may help in maintaining the physiological balance of autoimmunity and protective immunity by preserving the number of regulatory T cells and the activation of CD8^+^ T cells^53^. Similarly Zheng et al. also found that CD8^+^ T cells in severe patients were significantly lower than those in mild patients, suggesting that high levels of testosterone may reduce the incidence of severe disease by activating CD8^+^ T cells^54^. Despite the role of testosterone in modulating the immune response appears crucial, few data are available in this regard, and therefore more evidence is needed in order to correctly dispose of testosterone-targeted therapy^55^. Our systematic review and meta-analysis presents some limitations. Firstly, the descriptive nature of our analysis is unable to assess the causal direction in the relationship between testosterone levels and clinical outcomes. However, we believe that the analysis of other aspects such as LH levels and inflammatory markers adds important information regarding the association between testosterone and Covid-19 disease. According to our findings, we can speculate that low TT values are the result of a systemic inflammation process, but we cannot exclude that in the advanced stages of the disease low TT levels can play a crucial pathogenetic role dysregulating the immune response (Fig. 4). Secondly, we include studies from pre-print platforms which have not undergone peer review. However, given the descriptive nature of our research question (as opposed to a causal treatment effect), we assume that the risk of bias due to an absence of peer review process is low and, indeed, it may results in less publication bias. Our meta-analysis reveals diminished total testosterone (TT) levels in patients afflicted with Covid-19 infection, particularly in those experiencing more severe forms of the disease. Significantly, the normal luteinizing hormone (LH) levels observed both among cases and controls and across different disease stages seem to rule out primary hypogonadism as the underlying cause of the observed low testosterone values. The precise role of TT in modulating the immune response emerges as a relevant aspect that requires elucidation in future studies.

## Materials and methods

The current metanalysis has been registered on PROSPERO (N. CRD42022385459).

This systematic review and meta-analysis was conducted according to Preferred Reporting Items for Systematic Reviews and Meta-Analyses (PRISMA) guidelines and PRISMA Checklist was completed Suppl. Table 1^56^. A research question was established based on the Patient-Index Comparator-Outcome-Study design (PICOS) criteria using the following: what is the rate of hypogonadism in patients with Covid-19 infection? Is there a difference in the rate of hypogonadism according to the severity of Covid-19 disease? Furthermore, is there a difference in luteinizing hormone levels (LH) according to stage of Covid-19 disease? As a secondary aim, we explored the correlation between testosterone levels and some markers of inflammatory and immune response which have been considered relevant in the context of the Covid-19 pandemic.

### Evidence acquisition

A systematic literature search was performed on PubMed, Scopus, and Web of Science, ranging from pandemic inception to March 30th 2022. Additional searching was done in the *medRxiv* repository for relevant preprints. The following search strategies were used: testosterone OR androgens OR hypogonadism AND Covid-19 OR Severe Acute Respiratory Syndrome. All titles and abstracts were assessed to select studies reporting data on testosterone levels in patients with Covid-19 infection and matched controls. The references of the included studies were evaluated for other potential trials. PubMed “related articles” function was used to search for other studies. Titles and abstracts were independently screened by two reviewers (SS and MM). The full text of relevant articles was then reviewed to identify eligible studies. The Preferred Reporting Items for Systematic Review and Meta-analyses (PRISMA) was followed^56^.

### Selection of the studies and inclusion criteria

#### Inclusion criteria

- All controlled and observational studies which included patients who had testosterone levels and/or luteinizing hormone levels (LH) dosage at hospital admission.
- We included only studies in which a clear definition of disease’s stage or severity was reported according to the criteria of either world health organisation (WHO) or the National Health Commission of China.

#### Exclusion criteria

- We excluded studies that had fewer than 20 participants, or studies of which the measures of central tendency and distribution could not be obtained.
- Studies that did not report the results as a mean or median (e.g., total testosterone levels reported as frequencies of categorical concentrations, or in a figure that was difficult to interpret).

### Data extraction and data analysis

One of two reviewers (VC) extracted data from all studies that met inclusion criteria using a standardized data collection tool. The following data were reported: first authors, publication date, country of origin, groups for each stage of disease, cases and number of controls, age, TT and LH levels, levels of inflammatory markers such as interleukin 6 (IL-6), D-dimer, lymphocytes count, monocytes count, C-reactive protein (CRP) and frequency of clinical variables.

TT and LH were expressed as nmoL/L and mIU/mL, respectively. Therefore, studies reporting results in other unit of measurement were converted using validated tools. All included studies reported a measure of central tendency (mean or median) and dispersion (SD, standard error, IQR, or range) for testosterone and LH concentrations. Mean and SD were used when reported in selected studies. When median and IQR or range were reported, we adhered to Cochrane recommendations, estimating mean values using the method described by Wan and colleagues^57^ and the SD using the Cochrane handbook method^58^.

### Risk of bias assessment and statistical analysis

“Quality Assessment Tool for Observational Cohort and Cross- Sectional Studies” provided by the National Health Institute (NIH) was adopted to assess risk of bias (RoB) for each included studies^59^. Biases screened for included selection bias, information bias, measurement bias, or confounding bias (including cointerventions, differences at baseline in patient characteristics etc.). The quality of studies was rated as poor, fair, or good, with higher RoB leading to poor quality (“−”) ratings and low-RoB leading to good quality (“+”) ratings. Supp. Tab. 2 Publication bias was tested both by visual assessment of the Deeks’ funnel plot and calculation of p-value using the Deeks’ asymmetry test^60^. The ‘*Trim and Fill*’ method was implemented to explore the possible nature of studies ‘‘missed’’ in the review^61^. Statistical analyses along with reporting and interpretation of the results were conducted according to previously described methodology^62^ and consisted of several analytical steps.

Firstly, conventional meta-analysis of standard mean difference (SMD) and 95% confidence intervals (CIs) for serum TT and LH levels was performed by comparing the Covid-19 and matched controls populations using random effect model according to *DerSimonian–Laird* method^63^. This was additionally assessed at sub-groups levels by Covid-19 clinical severity in accordance with previously established Guidelines^64,65^. Sensitivity analyses were performed to assess the contribution of each study to the pooled estimate by excluding individual trials one at a time and recalculating the pooled estimates for the remaining studies (leave-one-out meta-analysis). Evaluation for presence of heterogeneity was done using the following^66^: (1) Cochran’s Q-test with *p* < 0.05 signifying heterogeneity; (2) Higgins I^2^ test with inconsistency index (I^2^) = 0–40%, heterogeneity might not be important; 30–60%, moderate heterogeneity; 50–90%, substantial heterogeneity; and 75–100%, considerable heterogeneity. Our results are graphically displayed as forest plots on a per-single study level, with pooled results indicating SMD for serum TT and LH levels. Subgroup analyses were performed looking at differences in categorical confounders (e.g., clustered study sample size, design, and year of publication). A cumulative meta-analysis was further performed to explore the trend in the effect sizes variation across subgroups as a function of the year of the Covid-19 pandemic. Meta-regression analyses were performed using available continuous variables retrieved among the studies. Pooled weighted estimates were plotted against the following clinical or haemato-chemical variables: IL-6 (pg/ml), total lymphocytes count, D-dimer, CRP; smoking status, hypertension; diabetes; chronic obstructive pulmonary disease; body mass index (BMI). Calculations were performed using the ‘*meta*’ package from Stata (Statistical Software: Release 17. College Station, TX: StataCorp LLC) with all tests being two sided, and statistical significance set at *p* < 0.05.

### Supplementary Information

Supplementary Information.

## Data Availability

The datasets used during the current study are available from the corresponding author on reasonable request.
